# Long-term follow-up of patients with intestinal neuronal dysplasia type B: Protocol for an observational, ambispective, and comparative study

**DOI:** 10.1097/MD.0000000000007485

**Published:** 2017-07-14

**Authors:** Pedro Luiz Toledo de Arruda Lourenção, Erika Veruska Paiva Ortolan, Laura Luiza Minelli Rosa, Marcos Curcio Angelini, Simone Antunes Terra, Maria Aparecida Marchesan Rodrigues

**Affiliations:** a Discipline of Pediatric Surgery—Department of Surgery and Orthopedics; b Botucatu Medical School, São Paulo State University (UNESP), São Paulo, Brazil; c Department of Pathology, Botucatu Medical School, São Paulo State University (UNESP), São Paulo, Brazil.

**Keywords:** intestinal chronic constipation, intestinal dysganglionosis, intestinal neuronal dysplasia type B

## Abstract

Intestinal neuronal dysplasia type B (IND-B) is a pathological entity of the group of gastrointestinal neuromuscular diseases characterized by complex alterations in the enteric nervous system. Patients typically present with intestinal constipation, sometimes complicated by episodes of intestinal obstruction. The 2 therapeutic modalities include conservative clinical treatment and surgical treatment. Nevertheless, the results of the different therapeutic modalities are conflicting, and follow-up studies are scarce and include only a limited number of patients.

This is a single-center, ambispective, observational, longitudinal, and comparative follow-up study to compare the results of conservative clinical and surgical treatments in patients with IND-B. Sixty-three patients (<15 years) who received this diagnosis will be included. These patients will be divided into 2 groups according to the type of treatment that they previously received: 29 patients in the surgical treatment group and 34 patients in the conservative treatment group. Previous data will be recovered from the medical records of the study patients, including signs and symptoms present at the time of diagnosis, particularly those related to bowel habits, and treatments undergone. Later, these patients will be invited to participate in a semistructured interview during which aspects related to the long-term functional results of the bowel habit and quality of life will be investigated after a minimum interval of 5 years posttreatment.

This project aims to assess the long-term clinical evolution of patients diagnosed with IND-B and compare the results obtained following conservative clinical and surgical treatments.

This protocol will provide sufficient data to analyze the long-term clinical outcome obtained through the 2 treatment modalities proposed for patients with IND-B.

## Abstract 2

Supplemental Digital Content is available in the text

## 1 Background

Intestinal neuronal dysplasia type B (IND-B) is a pathological entity of the group of gastrointestinal neuromuscular diseases characterized by complex alterations in the enteric nervous system.^[1]^ In 1971, Meier-Ruge^[2]^ described IND-B for the first time as a condition typically associated with distal intestinal obstruction that could mimic Hirschsprung disease (HD) but exhibited distinct histopathological characteristics. Since then, despite intensive scientific investigation, there remain inconsistencies with respect to the definition, etiopathogenesis, diagnostic criteria, and therapeutic possibilities of IND-B.^[3]^

IND-B is considered a rare disease, with an estimated incidence of 1:7500 live births.^[4]^ However, the frequency of IND-B varies widely, and the reported rates range from 0.3% to 40% of all rectal suction biopsies.^[5–8]^ The latest published series by Taguchi et al^[9]^ involved a retrospective multicenter study of IND-B cases in 167 centers in Japan between 2000 and 2009. These authors reported 13 cases based on standardized morphologic criteria from all of the included centers.^[9]^

The diagnosis of IND-B fundamentally depends on the histopathological analysis of rectum biopsies.^[10]^ However, the morphological criteria for its diagnosis have changed significantly over the years, rendering both diagnostic practice and study comparison difficult. Hyperplasia of the submucous nervous plexus is the morphological finding that defines IND-B, but specific morphological criteria may differ widely.^[2,11–14]^

Patients typically present with intestinal constipation, sometimes complicated by episodes of intestinal obstruction.^[3,15]^ In some cases, these symptoms may begin in the first days of life with a delay in meconium elimination, abdominal distension, vomiting, and feeding difficulties. Some patients continue to experience these symptoms throughout their lives, typically exhibiting severe intestinal constipation that is refractory to several types of treatments.^[16–18]^

The 2 therapeutic modalities include conservative clinical treatment and surgical treatment.^[19]^ Conservative treatment is based on changes in diet and the use of laxatives and enemas in cases of fecal impaction.^[3,20]^ Surgical treatment, on the other hand, may include sphincterotomy, colonic resection, or temporary colostomy.^[21–24]^ Nevertheless, the results of the different therapeutic modalities are conflicting, and follow-up studies are scarce and include only a limited number of patients. Most of these studies focus on only one modality of treatment and include short-term clinical follow-up. Therefore, only limited scientific evidence is available to establish a protocol to treat IND-B. However, in medical practice, we continue to identify children with severe intestinal constipation or bowel obstruction who undergo diagnostic investigation for HD with biopsies of the rectum that reveal submucosa nervous system hyperplasia compatible with the diagnosis of IND-B that require specific treatment.^[19]^ Therefore, we decided to assess the long-term clinical evolution of IND-B patients and compare the results obtained following conservative clinical and surgical treatments.

## 2 Methods/design

### 2.1 Study design and setting

This is a single-center, ambispective, observational, longitudinal, and comparative follow-up study to compare the results of conservative clinical and surgical treatments in patients with IND-B.

This study will be conducted at the Botucatu Medical School, São Paulo State University (UNESP), São Paulo, Brazil. Previous data will be recovered from the medical records of the study patients, including signs and symptoms present at the time of IND-B diagnosis, particularly those related to bowel habits, and treatments undergone. Later, these patients will be invited to participate in a semistructured interview during which aspects related to the long-term functional results of the bowel habit and quality of life will be investigated after a minimum interval of 5 years posttreatment.

### 2.2 Ethics approval and consent to participate

This study will be conducted in accordance with the principles of the Declaration of Helsinki, ISO14155, Data Protection Act, and the Guidelines for Good Clinical Practice. The Research Ethics Committee (REC) of the Botucatu Medical School, UNESP, São Paulo, Brazil, has approved this study, which was registered under number 11520712.6.0000.5411 (see REC, Supplemental Digital Content 1). The patients and/or their guardians were previously informed of the purpose of the research, and each signed an informed consent form (ICF) (see ICF, Supplemental Digital Content 2). Patients aged 11 to 18 years signed the respective specific consent form (SCF) (see SCF, Supplemental Digital Content 3). All data will be sent to the REC at the end of the study. The subjects may leave this study at any point in time without any limitations.

The Brazilian Registry of Clinical Trials (Rebec) identifier for this study is RBR-8r3b7y, obtained on September 28, 2016 (UTN Number: U1111-1185-4950), available at http://www.ensaiosclinicos.gov.br.

### 2.3 Eligibility criteria

The inclusion and exclusion criteria are presented in Table 1.

**Table 1:**
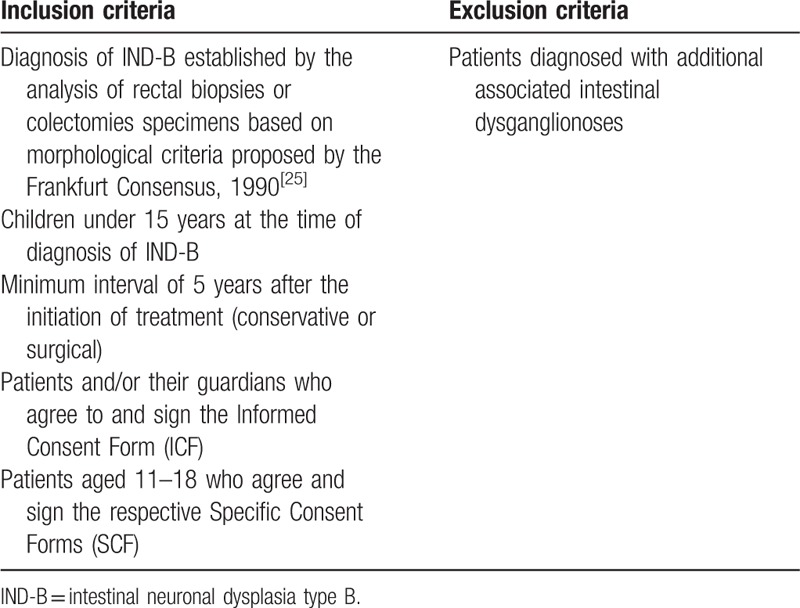
Eligibility criteria.

### 2.4 Patient selection, inclusion in treatment groups and recruitment

Sixty-three patients (<15 years) who received a diagnosis of IND-B at the University Hospital of Botucatu Medical School—UNESP between 1998 and 2012 will be included. The diagnosis of IND-B must have been established based on histopathological analysis of rectal biopsies or surgical specimens according to the morphological criteria proposed by the Frankfurt Consensus, 1990, with no additional associated dysganglionoses. These patients will be divided into 2 groups according to the type of treatment that they previously received: 29 patients in the surgical treatment group and 34 patients in the conservative treatment group.

The patients will be recruited through letters and phone calls and will be invited to attend the Clinical Research Unit (UPECLIN) of Botucatu Medical School—UNESP to participate in a semistructured interview.

### 2.5 Pretreatment details (retrospective analysis)

Previous data will be recovered from patient medical records, including the age at which the symptoms appeared, age at the time of diagnosis, gender, gestational age and weight at birth, clinical evolutions during the neonatal period and the presence of associated malformations. The signs and symptoms, particularly those related to bowel habits, present at the time of IND-B diagnosis (before beginning treatment) and the treatments undergone will also be recovered. The following clinical information will be retrieved: number of evacuations per week, number of episodes of fecal incontinence per week, presence of retentive posturing during defecation, straining to pass stool, pain with defecation, presence of fecaloma, presence of abdominal distension, presence of bloody stools, presence of abdominal pains, and the need for enemas.

### 2.6 Posttreatment data (clinical interviews)

**Figure 1:**
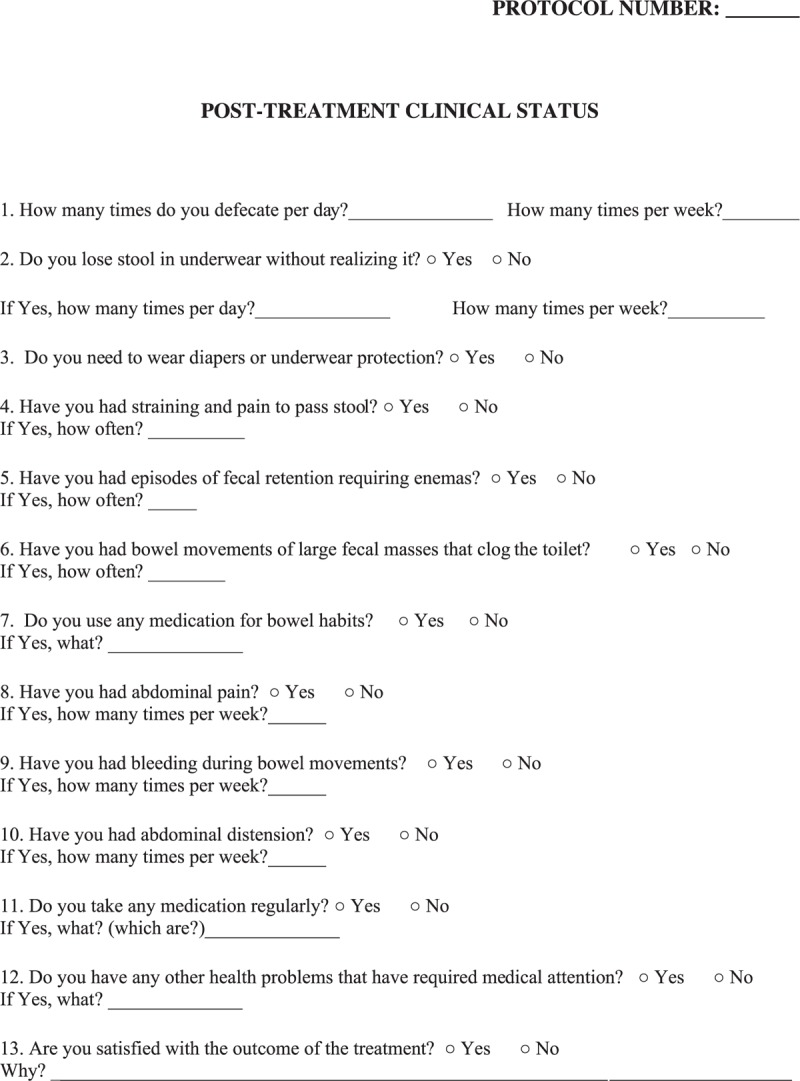
Questionnaire addressing the current clinical status.

Patients will participate in semistructured interviews to determine the functional results of the intervention with respect to patient bowel habits and quality of life after a minimum 5 years since the initiation of treatment. These interviews will be conducted by 2 pediatric surgeon members of the research team and will last approximately 40 minutes. The following assessment instruments will be applied: a questionnaire addressing current clinical status (Fig. 1), the Templeton & Ditesheim Scoring System (TDSS)^[26]^ to evaluate fecal incontinence (see TDSS, Supplemental Digital Content 4), the modified Bristol Stool Form Scale for Children (mBSFS-C)^[27,28]^ to analyze the consistency of feces (see mBSFS-C, Supplemental Digital Content 5), and the Pediatric Quality of Life Inventory version 4.0 (PEDsQL 4.0)^[29,30]^ to assess global quality of life (see PEDsQL 4.0, Supplemental Digital Content 6).

### 2.7 Outcomes

#### 2.7.1 Posttreatment clinical status

**Figure 2:**
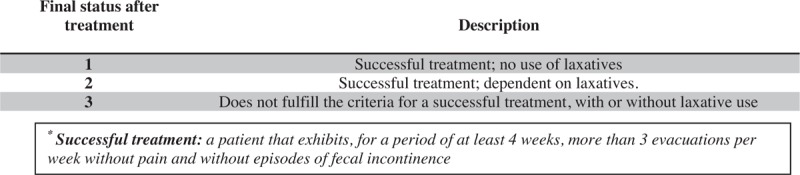
Classification of final status after treatment. Adapted from Tran et al^[31]^.

The primary evaluation of the long-term clinical evolution of IND-B patients will be based on posttreatment data obtained during the clinical interviews. The analysis will be performed according to a classification of clinical prognosis as proposed by Tran et al^[31]^ (Fig. 2). In addition, the functional bowel habits assessed by the TDSS^[26]^ and mBSFS-C^[27,28]^ and quality of life assessed by the PEDsQL 4.0^[29,30]^ will be evaluated.

#### 2.7.2 Evaluation before and after treatment

**Figure 3:**
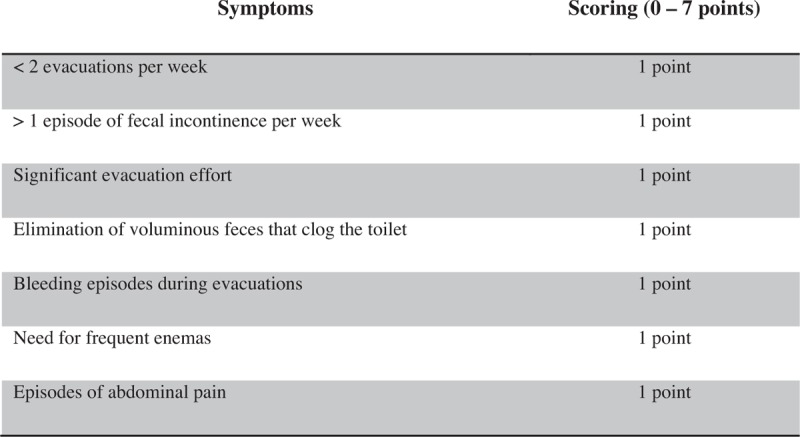
Intestinal Symptom Index.

Data analysis will be performed by focusing on 2 assessment time points: assessment at diagnosis before the initiation of treatment and assessment at the time of interview, after a minimum period of 5 years after the treatment. This analysis will be performed using the following clinical variables: number of evacuations per week, number of episodes of fecal incontinence per week, presence of retentive posturing during defecation, straining and pain to pass stool, presence of fecaloma, presence of abdominal distension, presence of bloody stools, presence of abdominal pains, and need for enemas. In addition, based on the clinical information obtained in these 2 assessments, we will apply the Bowel Function Score (BF-S)^[32–34]^ (see BF-S, Supplemental Digital Content 7) and a proposed Intestinal Symptom Index (Fig. 3), with a variance from 0 to 7, which will identify the most common clinical complaints and symptoms during the clinical course of IND-B.

#### 2.7.3 Comparison between treatment modalities

**Figure 4:**
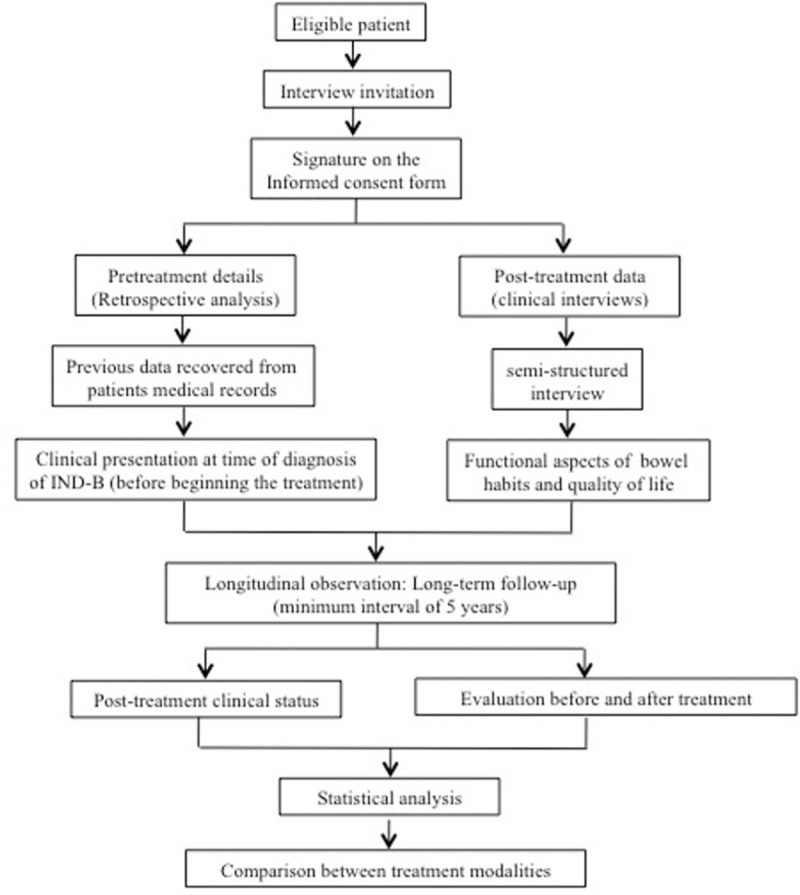
Flowchart of patients in the study.

The results obtained by the 2 treatment modalities (conservative clinical and surgical) will be compared with respect to the variables analyzed at both time points (before and after treatment) and with respect to the functional status and quality-of-life assessed after a long follow-up period. To summarize the stages of the study, see the flow diagram in Fig. 4 and the Supplemental Digital Content 8 with Standard Protocol Items: Recommendations for Interventional Trials (SPIRIT).

### 2.8 Statistical analysis

A statistical and descriptive analysis will be performed to interpret the clinical and demographic characteristics of the patients at the 2 time points of assessment. Continuous numerical data will be expressed as the mean ± standard deviation and median (minimum/maximum). Proportions will be presented as percentages and their respective reliability intervals. The comparison between the 2 treatment groups will be made using different statistical tests according to the types of variables analyzed. Nominal variables will be analyzed using Fisher exact test, and the Mann–Whitney *U* test will be used to analyze ordinal variables. The comparison between proportions will be made using the Binomial test. Continuous numerical variables of nonparametric distribution will be assessed using the Mann–Whitney *U* test. The analysis of global quality of life, assessed by means of the PEDsQL 4.0 questionnaire,^[29,30]^ will be compared to data previously published for a healthy pediatric population using the *t* test for differences between means. The significance level will be established at 5%, and the analysis will be performed using SPSS 22.0 for Windows.

### 2.9 Protocol amendments

Any amendments to the protocol and information provided to participants will be submitted to the REC for approval before implementation. Substantial amendments may only be implemented after REC approval has been obtained, whereas nonsubstantial amendments can be implemented without written approval from the REC. Data and source documents will be stored such that they can be accessed at a later date for monitoring or inspection by the REC. After the end of the study, the results from the trial will be submitted for publication in a peer-reviewed journal, following STROBE Compliant criteria. Authorship of any related presentations or reports will be under the name of the collaborative group.

## 3 Discussion

Few studies have reported the experience of IND-B patients, and most studies have focused on only one modality of treatment and included only short-term clinical follow-ups.^[17,18,21,35,36]^ Conservative clinical treatment must obey the general principles used in the treatment of children with chronic constipation and is focused on fecal disimpaction and laxatives at appropriate doses.^[37]^ Although it does not have a definite role like that in HD, surgical treatment may also be performed in IND-B patients.^[35]^ However, the surgical techniques used and the resected intestine segments vary significantly.^[15,19]^

Because IND-B is considered a rare disease, the proposed sample of 63 patients is considered satisfactory. Furthermore, one strength of this project is the possibility of comparing the results of the 2 treatment modalities proposed for patients with IND-B. This study is in the recruitment phase, which started in March 2015 and is still ongoing.

## Acknowledgments

The authors thank the other members of the research team who will participate in the application of this research protocol: Vanessa Mello Granado Cassettari, Alana Maia e Silva, Tainara Francini Felix, and Patricia Pintor dos Reis.

## Supplementary Material

Supplemental Digital Content
