# Supplementary material for: Long-term follow-up of patients with intestinal neuronal dysplasia type B: Protocol for an observational, ambispective, and comparative study
Source: Medicine (Baltimore). 2017 Jul 14;96(28):e7485. doi: 10.1097/MD.0000000000007485 (PMC5515763; doi:10.1097/MD.0000000000007485)
Supplement: Supplemental Digital Content [file medi-96-e7485-s001.pdf]

FACULDADE DE MEDICINA DE BOTUCATU -UNESP

PARECER CONSUBSTANCIADO DO CEP

DADOS DO PROJETO DE PESQUISA

Título da Pesquisa: Seguimento clínico a longo prazo de crianças com displasia neuronal intestinal
Pesquisador: Pedro Luiz Toledo de Arruda Lourenção
Área Temática:
Versão: 2
CAAE: 11520712.6.0000.5411
Instituição Proponente: Hospital das Clínicas da Faculdade de Medicina de Botucatu ((HCFMB))
Patrocinador Principal: Financiamento Próprio

DADOS DO PARECER

Número do Parecer: 211.156
Data da Relatoria: 04/03/2013

Apresentação do Projeto:
Área Temática Grupo III. Área do Conhecimento: 4.01 - Ciências da Saúde
Autor: Pedro Luiz Toledo de Arruda Lourenção
Departamento de Cirurgia e Ortopedia da FMB
Instituição: Hospital das Clínicas da Faculdade de Medicina de Botucatu
Objetivo acadêmico: não envolve obtenção de título acadêmico

Objetivo da Pesquisa:
Avaliar qual modalidade terapêutica, clínica ou cirúrgica, obteve melhores resultados a longo prazo nas crianças com displasia neuronal intestinal.
Obter informações sobre o curso clínico da doença que ainda não está bem estabelecido

Avaliação dos Riscos e Benefícios:
Não haverá prejuízos ou danos aos entrevistados. Os dados levantados serão retrospectivos e será realizada uma consulta (entrevista) com o paciente.
Benefícios: informações quanto aos sinais e sintomas dos pacientes no momento do diagnóstico e ao longo dos anos, que podem contribuir para o entendimento do curso clínico da doença, ainda pouco compreendido.
Obtenção de dados nacionais uma vez que a maior parte das investigações sobre a doença é proveniente do continente Europeu.

Comentários e Considerações sobre a Pesquisa:

Conforme parecer anterior, foi solicitado ao autor:

- Declaração de autorização de manipulação de arquivos de laudos, livro de registro de biópsias.
- Termo de assentimento (para maiores de 11 anos)
- Refazer o TCLE informando como seria a entrevista (consulta médica, o que seria feito na consulta, quem faria a consulta, como seriam realizados os registros médicos e se fossem encontrados problemas como seria feito o encaminhamento do paciente).

Demais quesitos já encontravam-se em ordem.

Considerações sobre os Termos de apresentação obrigatória:

O autor apresenta Declaração de autorização de manipulação de arquivos de laudos, livro de registro de biópsias devidamente assinado pela chefia do Serviço.

- Foi elaborado o termo de assentimento a ser aplicado nos maiores que 11 anos, com as devidas sugestões, assim como foram realizadas as alterações sugeridas no TCLE.

Recomendações:

Nenhuma

Conclusões ou Pendências e Lista de Inadequações:

Considero o projeto aprovado sem necessidade de envio a CONEP.

Situação do Parecer:

Aprovado

Necessita Apreciação da CONEP:

Não

Considerações Finais a critério do CEP:

Projeto de Pesquisa aprovado em reunião do CEP de 04 de março de 2.013, sem necessidade de envio à CONEP.

BOTUCATU, 05 de Março de 2013

Assinador por:
Trajano Sardenberg
(Coordenador)

TERMO DE CONSENTIMENTO LIVRE E ESCLARECIDO

Você e seu filho (a) estão sendo convidados (as) a participarem como voluntários (as) da pesquisa: “**Seguimento clínico em longo prazo de crianças com Displasia Neuronal Intestinal”.** O motivo que nos leva a estudar esta doença é a falta de informações sobre a resposta dos pacientes aos tratamentos empregados, após um longo período de tempo. Isto é importante para analisarmos qual foi a modalidade terapêutica que teve melhor resultado nos pacientes com diagnóstico de Displasia Neuronal Intestinal e, desta forma, melhorarmos a tratamento das crianças acometidas por esta doença. Nesta pesquisa serão levantadas as crianças com esta doença que foram tratadas em nosso hospital, no período de 1998 a 2009. Através da pesquisa dos dados dos prontuários destas crianças, nós vamos saber qual foi o tratamento realizado e como é que elas estão hoje em dia. Além disso, estas crianças serão convocadas e passarão por uma consulta médica, na qual elas ou seus responsáveis apenas responderão algumas perguntas para o médico, principalmente relacionadas ao hábito intestinal, e serão submetidas a um exame físico geral, no qual serão medidos o peso e a altura. Esta consulta médica será realizada por um médico cirurgião pediátrico e terá duração aproximada de 15 minutos. Os dados obtidos (respostas e resultados do exame físico) serão transcritos para um formulário eletrônico. Você acompanhará seu filho (a) em todos os momentos da consulta. Se durante esta consulta médica for diagnostico algum problema na saúde da criança, relacionada ou não à doença em investigação, será realizado o encaminhamento adequado da criança ao médico especialista responsável.

Você será esclarecido (a) sobre a pesquisa em qualquer aspecto que desejar. Você é livre para recusar-se a participar, retirar seu consentimento ou interromper a participação a qualquer momento. A sua participação é voluntária e a recusa em participar não irá acarretar qualquer penalidade ou perda de benefícios. Os pesquisadores irão tratar a identidade da criança com padrõesprofissionais de sigilo. Seu nome e o de seu filho (a) ou material que indique a sua participação não será liberado sem a sua permissão. Você ou seu filho (a) não será identificado(a) em nenhuma publicação que possa resultar deste estudo. Uma cópia deste consentimento informado será arquivada no Departamento de Cirurgia e Ortopedia da Faculdade de Medicina de Botucatu – Unesp. A participação no estudo não acarretará custos para você e não será disponível nenhuma compensação financeira adicional.

Certificado de Consentimento

Eu,____,responsável pelo (a) menor

____, fui informado (a) dos objetivos da pesquisa acima de maneira clara e detalhada e esclareci minhas dúvidas. Sei que em qualquer momento poderei solicitar novas informações e motivar minha decisão se assim o desejar. Declaro que concordo em participar desse estudo. Recebi uma cópia deste termo de consentimento livre e esclarecido e me foi dada a oportunidade de ler e esclarecer as minhas dúvidas.

Nome	Assinatura do Responsável	Data

Nome	Assinatura do Pesquisador	Data

Nome	Assinatura da Testemunha	Data

Em caso de dúvidas com respeito aos aspectos éticos deste estudo, você poderá consultar:

CEP- Comitê de Ética em Pesquisa da Faculdade de Medicina de Botucatu - Unesp

Chacára Butignoli s/n, Rubião Júnior - Botucatu - São Paulo - CEP: 18618-970

Telefones: (14) 3880-1608/3880-1609

Pesquisador Responsável: Pedro Luiz Toledo de Arruda Lourenção

Departamento de Cirurgia e Ortopedia da Faculdade de Medicina de Botucatu - Unesp

Avenida Prof. Montenegro, Distrito de Rubião Júnior, s/n - Botucatu - São Paulo – CEP 18.618-970 Telefones: (14) 3880- 1703

E-mail: lourencao@fmb.unesp.

TERMO DE ASSENTIMENTO

Você está sendo convidado (a) a participar da pesquisa "Seguimento clínico a longo prazo de crianças com Displasia Neuronal Intestinal". Neste estudo queremos saber mais sobre esta doença, chamada Displasia Neuronal Intestinal, que acomete o intestino de crianças e que ainda é pouco conhecida. Eu vou te informar sobre esta pesquisa e te convidar a participar conosco. Discutimos esta pesquisa com seus pais ou responsáveis e eles sabem que também estamos pedindo o seu acordo. Se você concordar em participar da pesquisa, seus pais ou responsáveis também terão que concordar. Mas se você não desejar fazer parte da pesquisa, não é obrigado, até mesmo se seus pais concordarem. Você pode discutir qualquer coisa deste formulário com seus pais, amigos ou qualquer um com quem você se sentir a vontade de conversar. Você pode decidir se quer participar ou não depois de ter conversado sobre a pesquisa e não é preciso decidir imediatamente. Pode haver algumas palavras que não entenda ou coisas que você quer que eu explique mais detalhadamente porque você ficou mais interessado ou preocupado. Por favor, peça para que eu pare a qualquer momento e assim eu te explicarei.

Através desta pesquisa nós queremos descobrir qual é o melhor tratamento para as crianças com esta doença. Para isto, nós vamos convocar todas as crianças que tiveram esta doença e foram tratadas em nosso hospital, no período de 1998 a 2009 e vamos pesquisar nos prontuários destas crianças qual foi o tratamento realizado e como é que elas estão hoje em dia. Além disso, estas crianças passarão por uma consulta médica, na qual apenas responderão algumas perguntas para o médico, principalmente relacionadas sobre o seu hábito intestinal, ou seja, como estão evacuando e passarão por um exame físico simples, na qual serão medidos o peso e altura.

Você está sendo convidado a participar desta pesquisa por fazer parte deste grupo de crianças, que passaram por tratamento para esta doença, neste hospital. Desta forma, esta pesquisa não oferece riscos adicionais ou desconforto a você, não sendo necessária a realização de nenhum procedimento invasivo, exame ou novo tratamento. Nada acontecerá além do que habitualmente ocorre em uma consulta médica, que deve durar aproximadamente 15 minutos. Participando desta pesquisa você estará ajudando na melhora do tratamento para as crianças com esta doença. Além disso, se durante este exame médico for descoberto algum problema em sua saúde, você será encaminhado ao médico especialista responsável.

Não falaremos para outras pessoas que você está nesta pesquisa e também não compartilharemos informação sobre você para qualquer um que não trabalha na pesquisa. Seu nome ou o material que indique sua participação não será liberado sem a permissão do responsável por você. Depois que a pesquisa acabar, os resultados serão informados para você e para seus pais. Você não precisa participar desta pesquisa se não quiser. É você quem decide. Se decidir não participar da pesquisa, é seu direito e nada mudará no seu tratamento de saúde. Mesmo assim, este serviço de saúde estará disponível para você. Até mesmo se você disser "sim" agora, poderá mudar de idéia depois, sem nenhum problema. Você pode me perguntar agora ou depois fazer as perguntas para outros membros da equipe da pesquisa. Eu escrevi um número de telefone e endereço onde você pode nos localizar ou, se você estiver por perto, você poderá vir e nos ver. Este termo de assentimento encontra-se impresso em duas vias, sendo que uma cópia será arquivada pelo pesquisador responsável, e a outra será fornecida a você.

Certificado do Assentimento

Eu entendi que a pesquisa é sobre uma doença chamada Displasia Neuronal Intestinal e que objetiva descobrir a melhor forma de tratamento para as crianças com esta doença. Eu entendi que participarei de uma consulta médica, respondendo algumas perguntas e passarei por um exame físico e que os dados do meu prontuário serão consultados. Sei que a qualquer momento poderei solicitar novas informações, e o meu responsável poderá modificar a decisão de participar se assim o desejar. Tendo o consentimento do meu responsável já assinado, declaro que concordo em participar desse estudo. Recebi uma cópia deste termo assentimento e me foi dada a oportunidade de ler e esclarecer as minhas dúvidas.

Assinatura da criança/adolescente:____

Assinatura dos pais/responsáveis:____

Assinatura do pesquisador: ____

Botucatu, ____ de _____________ de _______.

Em caso de dúvidas com respeito aos aspectos éticos deste estudo, você poderá consultar:

CEP- Comitê de Ética em Pesquisa da Faculdade de Medicina de Botucatu - Unesp

Chacára Butignoli s/n, Rubião Júnior - Botucatu - São Paulo - CEP: 18618-970

Telefones: (14) 3880-1608/3880-1609

Pesquisador Responsável: Pedro Luiz Toledo de Arruda Lourenção

Departamento de Cirurgia e Ortopedia da Faculdade de Medicina de Botucatu - Unesp

Avenida Prof. Montenegro, Distrito de Rubião Júnior, s/n - Botucatu - São Paulo – CEP 18.618-970

Telefones: (14) 3880- 1703 E-mail: lourencao@fmb.unesp.br

Quantitative assessment of fecal continence proposed by Templeton & Ditesheim, 1985

1. Toilet training for stool	
(A) Successful	1.0
(B) Occasionally successful (awareness of impending stool)	0.5
(C) No awareness of impending stool	0
2. Accidents	
(A) None, or rare	1.0
(B) 3 per week or less	0.5
(C) More than 3 per week	0
3. Extra underpants (or liners) needed	
(A) Never	1.0
(B) Only when having diarrhea	0.5
(C) Always	0
4. Social problems	
(A) None	1.0
(B) Infrequent odor; does not miss school, but no overnights, dates, camping, etc	0.5
(C) Frequent odor affects school and play	0
5. Activity restrictions	
(A) None	0.5
(B) Avoids swimming, sports, etc	0
6. Rashes	
(A) No current problems	0.5
(B) Some current problems	0
Total Score (Range)	0–5

*Good (4–5 points), fair (2–3.5 points), poor (0–1.5 points).

Templeton JM Jr, Ditesheim JA. High imperforate anus--quantitative results of long term fecal continence. J Pediatr Surg. 1985; 20(6):645-652.

modified Bristol Stool Form Scale for Children – m-BSFS-C

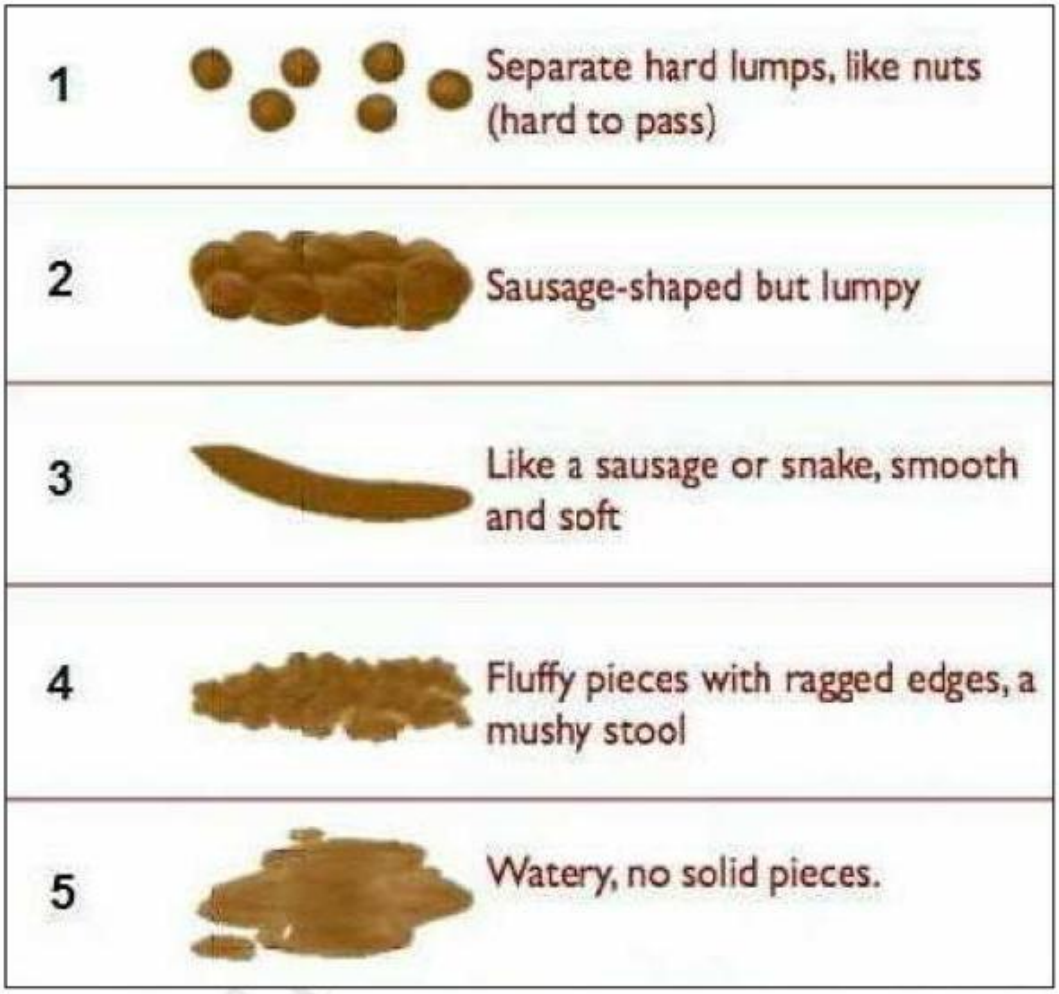

-Chumpitazi BP, Lane MM, Czyzewski DI, Weidler EM, Swank PR, Shulman RJ. Creation and initial evaluation of a stool form scale for children. J Pediatr. 2010;157: 594-597.

- Lane MM, Czyzewski DI, Chumpitazi BP, Shulman RJ. Reliability and validity of a modified Bristol Stool Form Scale for children. J Pediatr. 2011;159:437-441.

Mapi Research Trust

User agreement

Special Terms

Mapi Research Trust, a non-for-profit organisation subject to the terms of the French law of 1st July 1901, registered in Carpentras under number 453 979 346, whose business address is 27 rue de la Villette, 69003 Lyon, France, hereafter referred to as "Mapi" and the User, as defined herein, (each referred to singularly as a "Party" and/or collectively as the "Parties"), do hereby agree to the following User Agreement Special and General Terms:

Mapi Research Trust
Information Support Unit
27 rue de la Villette
69003 Lyon
France
Telephone: +33 (0)4 72 13 65 75
Fax: +33 (0)4 72 13 66 82
Email: PROinformation@mapi-trust.org

Recitals

The User acknowledges that it is subject to these Special Terms and to the General Terms of the Agreement, which are included in Appendix 1 to these Special Terms and fully incorporated herein by reference. Under the Agreement, the Questionnaire referenced herein is licensed, not sold, to the User by Mapi for use only in accordance with the terms and conditions defined herein. Mapi reserves all rights not expressly granted to the User.

The Parties, in these Special Terms, intend to detail the special conditions of their partnership.

The Parties intend that all capitalized terms in the Special Terms have the same definitions as those given in article 1 of the General Terms included in Appendix 1.

In this respect, the Parties have agreed as follows:

Article 1. Conditions Specific to the User

Section 1.01 Identification of the User

**User name**	Pedro Luiz Toledo de Arruda Lourenção
Legal Form	Pedro Luiz Toledo de Arruda Lourenção
Address	[São Paulo State University – UNESP, Botucatu Medical School]<br>Address : [Departamento de Cirurgia e Ortopedia – Anexo Verde<br>Faculdade de Medicina de Botucatu – Campus da Unesp Rubião Junior<br>Distrito de Rubião Junior, s/n – Botucatu, São Paulo, Brazil<br>ZIP code: 18618970, Brazil
Country	Brazil

**Name of the contact in charge of the Agreement**	
Telephone number	
Fax number	
Email address	lourencao@fmb.unesp.br
**If different:**	
Legal Form	
Address	
Country	

Section 1.02 Identification of the Questionnaire

Title	Pediatric Quality of Life Inventory™ (PedsQL™)
Author(s)	Varni James W, PhD

© Mapi Research Trust, December 1994, 2014. The unauthorized modification and use of any portion of this document is prohibited.

Owner	Varni James W, PhD
Copyright	Copyright © 1998 JW Varni, Ph.D. All rights reserved
Original bibliograhic references	See Appendix 2

Article 2. Rights to Use

Section 2.01 Context of the Use of the Questionnaire

The User undertakes to only use the Questionnaire in the context of the Study as defined hereafter.

**Context of use**	Clinical project or study
Title	Long term Follow up in patients with Intestinal Neuronal Dysplasia
Disease or condition	Neuronal Intestinal Dysplasia (NID)
Type of research	Epidemiologic/Observational
Number of patient expected	63
Number of submission to the Questionnaire for each patient	1
Term of clinical follow-up for each patient	
Mode of administration	Paper

Section 2.02 Conditions for Use

The User undertakes to use the Questionnaire in accordance with the conditions for use defined hereafter.

(a) Rights transferred
Acting in the Author's name, Mapi transfers the following limited, non-exclusive rights, to the User (the "Limited Rights")
(i) to use the Questionnaire, only as part of the Study; this right is made up exclusively of the right to communicate it to the Beneficiaries only, free of charge, by any means of communication and by any means of remote distribution known or unknown to date, subject to respecting the conditions for use described hereafter; and

(ii) to reproduce the Questionnaire, only as part of the Study; this right is made up exclusively of the right to physically establish the Questionnaire or to have it physically established, on any paper, electronic, analog or digital medium, and in particular documents, articles, studies, observations, medical publications, websites whether or not protected by restricted access, CD, DVD, CD-ROM, hard disk, USB flash drive, for the Beneficiaries only and subject to respecting the conditions for use described hereafter; and

(iii) Should the Questionnaire not already have been translated into the language requested, the User is entitled to translate the Questionnaire or have it translated in this language, subject to informing Mapi of the same beforehand by the signature of a Translation Agreement and to providing a copy of the translation thus obtained as soon as possible to Mapi.

The User acknowledges and accepts that it is not entitled to amend, condense, adapt, reorganise the Questionnaire on any medium whatsoever, in any way whatsoever, even minor, without Mapi's prior specific written consent.

(b) Specific conditions for the Author

The Author has intended to transfer a part of the copyright on the Questionnaire and/or the Documentation to Mapi in order to enable Mapi to make it available to the User for the purpose of the Study, subject to the User respecting the following conditions:

User shall not modify, abridge, condense, translate, adapt, recast or transform the Questionnaire in any manner or form, including but not limited to any minor or significant change in wordings or organisation in the Questionnaire, without the prior written agreement of the Author. If permission is granted, any improvements, modifications, or enhancements to the Questionnaire which may be conceived or developed, including translations and modules, shall become the property of the Author.

The User therefore undertakes to respect these special terms.

(c) Specific conditions for the Questionnaire

- Use in individual clinical practice or Research study / project

The User undertakes never to duplicate, transfer or publish the Questionnaire without indicating the Copyright Notice.

- In the case of use of an electronic version of the Questionnaire, the User undertakes to respect the following special obligations:

- Not modify the questionnaire (items and response scales, including the response scale numbers from 0-4)
- Cite the reference publications
- Insert the copyright notice on all pages/screens on which the Questionnaire will be presented and insert the Trademark information: PedsQL™, Copyright © 1998 JW Varni, Ph.D. All rights reserved.

© Mapi Research Trust, December 1994, 2014. The unauthorized modification and use of any portion of this document is prohibited.

- Mention the following information: "PedsQL™ contact information and permission to use: Mapi Research Trust, Lyon, France. E-mail: PROinformation@mapi-trust.org – Internet: www.proqolid.org and www.pedsql.org/index.html "
- Submit the screenshots of the US English original version of all the Pages where the Questionnaire appears to the Author, through Mapi Research Trust, before implementation in the translated versions and before release for approval and to check that the above-mentioned requirements have been respected.

• Use in a publication:

In the case of a publication, article, study or observation on paper or electronic format of the Questionnaire, the User undertakes to respect the following special obligations:

- not to include any full copy of the Questionnaire, but a version with the indication "sample copy, do not use without permission"
- to indicate the name and copyright notice of the author
- to include the reference publications of the Questionnaire
- to indicate the details of Mapi Research Trust for any information on the Questionnaire as follows: contact information and permission to use: Mapi Research Trust, Lyon, France. E-mail: PROinformation@mapi-trust.org – Internet: www.proqolid.org and www.pedsql.org
- to provide Mapi, as soon as possible, with a copy of any publication regarding the Questionnaire, for information purposes.

• Use for dissemination or marketing:

In the case of use in a dissemination/marketing context:

- On a website with unrestricted access:

In the case of publication on a website with unrestricted access, the User undertakes only to include a copy of the Questionnaire that cannot be amended, including the watermark on all pages or screens indicating "Sample copy – do not use without permission" along with the copyright notice and Mapi Research Trust's contact information.

- On a website with restricted access:

In the case of publication on a website with restricted access, the User may include a version of the Questionnaire that may be amended, subject to this version being protected by a sufficiently secure access to only allow the Beneficiaries to access it.

Article 3. Term

Mapi transfers the Limited Rights to use the Questionnaire as from the date of delivery of the Questionnaire to the User and for the whole period of the Study.

Article 4. Beneficiaries

The Parties agree that the User may communicate the Questionnaire in accordance with the conditions defined above to the Beneficiaries involved in the Study only, in relation to the Study defined in section 2.01.

Article 5. Territories and Languages

Mapi transfers the Limited Rights to use the Questionnaire on the following territories and in the languages indicated in the table below:

Language
Portuguese for Brazil

Versions/Modules		
PedsQL™ Gastrointestinal Symptoms Module	PedsQL™ Generic Core Scales	PedsQL™ Infant Scales

Article 6. Price and Payment Terms

The User undertakes in relation to Mapi to pay the price owed in return for the availability of the Questionnaire, according to the prices set out below, depending on the languages requested and the costs of using the Questionnaire, in accordance with the terms and conditions described in section 6.02 of the General Terms included in Appendix 1.

Access to the Questionnaire in non-funded academic research and individual clinical practice is free of charge.

Agreed and acknowledged by

User's name: Pedro Luiz Toledo de Arruda Lourenção

© Mapi Research Trust, December 1994, 2014. The unauthorized modification and use of any portion of this document is prohibited.

Date:
19/03/2014

Appendix 1 to the Special Terms: User Agreement General Terms

User has read and accepted the Mapi's General Terms of the Agreement, which are available on Mapi Research Trust website (http://www.mapi-trust.org/services/questionnairelicensing/catalog-questionnaires)

Appendix 2 to the Special Terms: References

Generic Core Scales:

- Varni JW, et al. The PedsQL™: Measurement Model for the Pediatric Quality of Life Inventory. *Medical Care*, 1999; 37(2):126-139

- Varni, J.W., et al. The PedsQL™ 4.0: Reliability and validity of the Pediatric Quality of Life Inventory™ Version 4.0 Generic Core Scales in healthy and patient populations. *Medical Care*, 2001; 39(8): 800-812.

- Varni, J.W., et al., (2002). The PedsQL™ 4.0 Generic Core Scales: Sensitivity, responsiveness, and impact on clinical decision-making. *Journal of Behavioral Medicine*, 25, 175-193.

- Varni, J.W., et al. (2003). The PedsQL™ 4.0 as a pediatric population health measure: Feasibility, reliability, and validity. *Ambulatory Pediatrics* , 3, 329-341.

- Chan, K.S., Mangione-Smith, R., Burwinkle, T.M., Rosen, M., & Varni, J.W. (2005). The PedsQL™: Reliability and validity of the Short-Form Generic Core Scales and Asthma Module. *Medical Care*, 43, 256-265.

- Varni, J.W., & Limbers, C.A. (2009). The PedsQL™ 4.0 Generic Core Scales Young Adult Version: Feasibility, reliability and validity in a university student population. *Journal of Health Psychology*, 14, 611-622.

Asthma Module:

- Varni, J.W., Burwinkle, T.M., Rapoff, M.A., Kamps, J.L., & Olson, N. The PedsQL™ in pediatric asthma: Reliability and validity of the Pediatric Quality of Life Inventory™ Generic Core Scales and Asthma Module. *Journal of Behavioral Medicine*, 2004; 27:297-318.

- Chan, K.S., Mangione-Smith, R., Burwinkle, T.M, Rosen, M., & Varni, J.W. (2005). The PedsQL™: Reliability and validity of the Short-Form Generic Core Scales and Asthma Module. *Medical Care*, 43, 256-265.

Brain Tumor Module:

- Palmer, S.N., Meeske, K.A., Katz, E.R., Burwinke, T.M., & Varni, J.W. (2007). The PedsQL™ Brain Tumor Module: Initial reliability and validity. *Pediatric Blood and Cancer*, 49, 287-293.

Cancer Module:

- Varni, J.W., Burwinkle, T.M., Katz, E.R., Meeske, K., & Dickinson, P. The PedsQL™ in pediatric cancer: Reliability and validity of the Pediatric Quality of Life Inventory™ Generic Core Scales, Multidimensional Fatigue Scale, and Cancer Module. *Cancer*, 2002; 94: 2090-2106.

- Robert RS, Paxton RJ, Palla SL, Yang G, Askins MA, Joy SE, Ater JL. Feasibility, reliability, and validity of the pediatric quality of life inventory™ generic core scales, cancer module, and multidimensional fatigue scale in long-term adult survivors of pediatric cancer. *Pediatric Blood & Cancer* 2012;59:703–707.

Cerebral Palsy Module:

- Varni JW, Burwinkle TM, Berrin SJ, Sherman SA, Artavia K, Malcarne VL, Chambers HG (2006). The PedsQL™ in Pediatric Cerebral Palsy: Reliability, Validity, and Sensitivity of the Generic Core Scales and Cerebral Palsy Module. *Developmental Medicine and Child Neurology*, 48: 442-449.

Cardiac Module:

- Uzark, K., Jones, K., Burwinkle, T.M., & Varni, J.W. The Pediatric Quality of Life Inventory™ in children with heart disease.Progress in Pediatric Cardiology, 2003; 18:141-148.

© Mapi Research Trust, December 1994, 2014. The unauthorized modification and use of any portion of this document is prohibited.

- Uzark, K., Jones, K., Slusher, J., Limbers, C.A., Burwinkle, T.M., & Varni, J.W. (2008). Quality of life in children with heart disease as perceived by children and parents. *Pediatrics*, 121, e1060-e1067.

Cognitive Functioning Scale:

- McCarthy, M.L., MacKenzie, E.J., Durbin, D.R., Aitken, M.E., Jaffe, K.M., Paidas, C.N. et al. (2005). The Pediatric Quality of Life Inventory: An evaluation of its reliability and validity for children with traumatic brain injury. *Archives of Physical Medicine and Rehabilitation*, 86, 1901-1909.

- Varni, J.W., Burwinkle, T.M., Katz, E.R., Meeske, K., & Dickinson, P. (2002). The PedsQL™ in pediatric cancer: Reliability and validity of the Pediatric Quality of Life Inventory™ Generic Core Scales, Multidimensional Fatigue Scale, and Cancer Module. *Cancer*, 94, 2090-2106.

- Varni, J.W., Limbers, C.A., Sorensen, L.G., Neighbors, K., Martz, K., Bucuvalas, J.C., & Alonso, E.M. (2011). PedsQL™ Cognitive Functioning Scale in pediatric liver transplant recipients: Feasibility, reliability and validity. *Quality of Life Research*, 20, 913–921.

Diabetes Module:

- Varni, J.W., Curtis, B.H., Abetz, L.N., Lasch, K.E., Piault, E.C., & Zeytoonjian, A.A. (in press). Content validity of the PedsQL™ 3.2 Diabetes Module in newly diagnosed patients with Type 1 Diabetes Mellitus ages 8-45. *Quality of Life Research*.

- Varni, J.W., Burwinkle, T.M., Jacobs, J.R., Gottschalk, M., Kaufman, F., & Jones, K.L. The PedsQL™ in Type 1 and Type 2 diabetes: Reliability and validity of the Pediatric Quality of Life Inventory™ Generic Core Scales and Type 1 Diabetes Module. *Diabetes Care*, 2003; 26: 631-637.

- Nansel, T.R., Weisberg-Benchell, J., Wysocki, T., Laffel, L. & Anderson, B. (2008). Quality of life in children with Type 1 diabetes: A comparison of general and disease-specific measures and support for a unitary diabetes quality of life construct. *Diabetic Medicine*, 25, 1316-1323.

- Naughton, M.J., Ruggiero, A.M., Lawrence, J.M., Imperatore, G., Klingensmith, G.J. Waitzfelder, B., McKeown, R.E., Standiford, D.A., Liese, A.D., & Loots, B. (2008). Health-related quality of life of children and adolescents with type 1 or type 2 diabetes mellitus: SEARCH for Diabetes in Youth Study. *Archives of Pediatrics and Adolescent Medicine*, 162, 649-657.

- Hilliard, M.E., Lawrence, J.M., Modi, A.C., Anderson, A., Crume, T., Dolan, L.M., Merchant, A.T., Yi-Frazier, J.P., & Hood, K.K. (2013). Identification of minimal clinically important difference scores of the Pediatric Quality of Life Inventory in children, adolescents, and young adults with Type 1 and Type 2 diabetes. *Diabetes Care*, 36, 1891–1897.

Duchenne Muscular Dystrophy Module:

- Uzark, K., King, E., Cripe, L., Spicer, R., Sage, J., Kinnett, K., Wong, B., Pratt, J., & Varni, J.W. (2012). Health-related quality of life in children and adolescents with Duchenne Muscular Dystrophy. *Pediatrics*, 130, e1559-e1566.

End Stage Renal Disease Module:

- Goldstein, S.L., Graham, N., Warady, B.A., Seikaly, M., McDonald, R., Burwinkle, T.M., Limbers, C.A., & Varni, J.W. (2008). Measuring health-related quality of life in children with ESRD: Performance of the Generic and ESRD-Specific Instrument of the Pediatric Quality of Life Inventory™ (PedsQL™). *American Journal of Kidney Diseases*, 51, 285-297.

Eosinophilic Esophagitis:

- Franciosi, J.P., Hommel, K.A., Bendo, C.B., King, E.C., Collins, M.H., Eby, M.D., Marsolo, K., Abonia, J.P., von Tiehl, K.F., Putnam, P.E., Greenler, A.J., Greenberg, A.B., Bryson, R.A., Davis, C.M., Olive, A.P., Gupta, S.K., Erwin, E.A., Klinnert, M.D., Spergel, J.M., Denham, J.M., Furuta, G.T., Rothenberg, M.E., & Varni, J.W. (2013). PedsQL™ Eosinophilic Esophagitis Module: Feasibility, reliability and validity. *Journal of Pediatric Gastroenterology & Nutrition*, 57, 57-66.

- Franciosi, J.P., Hommel, K.A., Greenberg, A.B., Debrosse, C.W., Greenler, A.J., Abonia, J.P., Rothenberg, M.E., & Varni, J.W. (2012). Development of the Pediatric Quality of Life Inventory™ Eosinophilic Esophagitis Module items: Qualitative methods. *BMC Gastroenterology*, 12:135, 1-8.

- Franciosi J.P., Hommel, K.A., Debrosse, C.W., Greenberg, A.B., Greenler, A.J., Abonia, J.P., Rothenberg, M.E., & Varni, J.W. (2012). Quality of life in paediatric eosinophilic oesophagitis: What is important to patients? *Child: Care, Health and Development*, 38, 477–483.

Family Impact Module:

- Varni, J.W., Sherman, S.A., Burwinkle, T.M., Dickinson, P.E., & Dixon, P. (2004). The PedsQL™ Family Impact Module: Preliminary reliability and validity. *Health and Quality of Life Outcomes*, 2 (55), 1-6.

- Medrano, G.R., Berlin, K.S., & Davies, W.H. (in press). Utility of the PedsQL™ Family Impact Module: Assessing the psychometric properties in a community sample. *Quality of Life Research*.

- Jiang, X., Sun, L., Wang, B., Yang, X., Shang, L., & Zhang, Y. (2013). Health-related quality of life among children with recurrent respiratory tract infections in Xi'an, China. *PLoS One*, 8(2): e56945.

© Mapi Research Trust, December 1994, 2014. The unauthorized modification and use of any portion of this document is prohibited.

- Mano, K.E., Khan, K.A., Ladwig, R.J., & Weisman, S.J. (2011). The impact of pediatric chronic pain on parents' health-related quality of life and family functioning: Reliability and validity of the PedsQL 4.0 Family Impact Module. *Journal of Pediatric Psychology*, 36, 517-527.

Gastrointestinal Symptom Scale:

- Varni, J.W., Lane, M.M., Burwinkle, T.M., Fontaine, E.N., Youssef, N.N., Schwimmer, J.B., Pardee, P.E., Pohl, J.F., & Easley, D.J. (2006). Health-related quality of life in pediatric patients with irritable bowel syndrome: A comparative analysis. *Journal of Developmental and Behavioral Pediatrics*,27, 451-458.

General Well-Being Scale:

- Varni, J.W., Seid, M., & Kurtin, P.S. (1999). Pediatric health-related quality of life measurement technology: A guide for health care decision makes. *Journal of Clinical Outcomes Management*, 6, 33-40.

- Hallstrand, T.S., Curtis, J.R., Aitken, M.L., & Sullivan, S.D. (2003). Quality of life in adolescents with mild asthma. *Pediatric Pulmonology*, 36, 536-543.

Healthcare Satisfaction Generic Module:

- Varni, J.W., Burwinkle, T.M., Dickinson, P., Sherman, S.A., Dixon, P., Ervice, J.A., Leyden, P.A. & Sadler, B.L. (2004). Evaluation of the built environment at a Children's Convalescent Hospital: Development of the Pediatric Quality of Life Inventory™ Parent and Staff Satisfaction Measures for pediatric health care facilities. *Journal of Developmental and Behavioral Pediatrics*, 2004; 25:10-25.

Health Care Satisfaction Module specific for Hematology/Oncology:

- Varni, J.W., Quiggins, D.J.L., & Ayala, G.X. (2000). Development of the Pediatric Hematology/Oncology Parent Satisfaction survey. *Children's Health Care*, 29, 243-255.

Infant Scales:

- Varni, J.W., Limbers, C.A., Neighbors, K., Schulz, K., Lieu, J.E.C., Heffer, R.W., Tuzinkiewicz, K., Mangione-Smith, R., Zimmerman, J.J., & Alonso, E.M. (2011). The PedsQL™ Infant Scales: Feasibility, internal consistency reliability and validity in healthy and ill infants. *Quality of Life Research*, 20, 45-55.

Multidimensional Fatigue Scale:

- Varni, J.W., Burwinkle, T.M., Katz, E.R., Meeske, K., & Dickinson, P. (2002). The PedsQL™ in pediatric cancer: Reliability and validity of the Pediatric Quality of Life Inventory™ Generic Core Scales, Multidimensional Fatigue Scale, and Cancer Module. *Cancer*, 94, 2090-2106.

- Varni, J. W., Beaujean, A., & Limbers, C. A. (in press). Factorial invariance of pediatric patient self-reported fatigue across age and gender: A multigroup confirmatory factor analysis approach utilizing the PedsQL™ Multidimensional Fatigue Scale. *Quality of Life Research*.

- Varni, J.W., Burwinkle, T.M., & Szer, I.S. (2004). The PedsQL™ Multidimensional Fatigue Scale in pediatric rheumatology: Reliability and validity. *Journal of Rheumatology*, 31, 2494-2500.

- Varni, J.W., & Limbers, C.A. (2008). The PedsQL™ Multidimensional Fatigue Scale in young adults: Feasibility, reliability and validity in a university student population. *Quality of Life Research*, 17, 105-114.

Neurofibromatosis Type 1 Module:

- Nutakki, K., Hingtgen, C.M., Monahan, P., Varni, J.W., & Swigonski, N.L. (2013). Development of the adult PedsQL™ Neurofibromatosis Type 1 Module: Initial feasibility, reliability and validity. *Health and Quality of Life Outcomes*, 11:21, 1-9

Neuromuscular Module:

- Iannaccone, S.T., Hynan, L.S., Morton, A., Buchanan, R., Limbers, C.A., & Varni, J.W. (2009). The PedsQL™ in pediatric patients with Spinal Muscular Atrophy: Feasibility, reliability, and validity of the Pediatric Quality of Life Inventory™ Generic Core Scales and Neuromuscular Module. *Neuromuscular Disorders*, 19, 805-812.

- Davis, S.E., Hynan, L.S., Limbers, C.A., Andersen, C.M., Greene, M.C., Varni, J.W., & Iannaccone, S.T. (2010). The PedsQL™ in pediatric patients with Duchenne Muscular Dystrophy: Feasibility, reliability, and validity of the Pediatric Quality of Life Inventory™ Neuromuscular Module and Generic Core Scales. *Journal of Clinical Neuromuscular Disease*, 11, 97-109.

Oral Health Scale:

© Mapi Research Trust, December 1994, 2014. The unauthorized modification and use of any portion of this document is prohibited.

- Steele, M.M., Steele, R.G., & Varni, J.W. (2009). Reliability and validity of the PedsQL™ Oral Health Scale: Measuring the relationship between child oral health and health-related quality of life. *Children's Health Care*, 38, 228-224.

Pediatric Pain Coping Inventory™:

- Varni, J.W., Waldron, S.A., Gragg, R.A., Rapoff, M.A., Bernstein, B.H., Lindsley, C.B., & Newcomb, M.D (1996). Development of the Waldron/Varni Pediatric Pain Coping Inventory. *Pain*, 67, 141-150.

Pediatric Pain Questionnaire:

- Varni, J.W., Thompson, K.L., & Hanson, V. (1987). The Varni/Thompson Pediatric Pain Questionnaire: I. Chronic musculoskeletal pain in juvenile rheumatoid arthritis. *Pain*, 28, 27-38.

Present Functioning Visual Analogue Scales:

- Sherman, S.A., Eisen, S., Burwinkle, T.M., & Varni, J.W. (2006). The PedsQL™ Present Functioning Visual Analogue Scales: Preliminary reliability and validity. *Health and Quality of Life Outcomes*, 4:75, 1-10.

Sickle Cell Disease Module:

- Panepinto, J.A., Torres, S., Bendo, C.B., McCavit, T.L., Dinu, B., Sherman-Bien, S., Bemrich-Stolz, C., & Varni, J.W. (2013). PedsQL™ Sickle Cell Disease Module: Feasibility, reliability and validity. *Pediatric Blood & Cancer*, 60, 1338–1344.

- Panepinto, J.A., Torres, S., & Varni, J.W. (2012). Development of the PedsQL™ Sickle Cell Disease Module Items: Qualitative methods. *Quality of Life Research*, 21, 341-357.

Rheumatology Module:

- Varni, J.W., Seid, M., Knight, T.S., Burwinkle, T.M., Brown, J., & Szer, I.S. (2002). The PedsQL™ in pediatric rheumatology: Reliability, validity, and responsiveness of the Pediatric Quality of Life Inventory™ Generic Core Scales and Rheumatology Module. *Arthritis and Rheumatism*, 2002; 46: 714-725.

Transplant Module:

- Weissberg-Benchell, J., Zielinski, T.E., Rodgers, S., Greenley, R.N., Askenazi, D., Goldstein, S.L., Fredericks, E.M., McDiarmid, S., Williams, L., Limbers, C.A., Tuzinkiewicz, K., Lerret, S., Alonso, E.M., & Varni, J.W. (2010). Pediatric health-related quality of life: Feasibility, reliability and validity of the PedsQL™ Transplant Module. *American Journal of Transplantation*, 10, 1677-1685.

© Mapi Research Trust, December 1994, 2014. The unauthorized modification and use of any portion of this document is prohibited.

N° de identificação: ______________

Data: ______________________

PedsQL™

Questionário sobre qualidade de vida para adultos jovens

Version 4.0 – Portuguese for Brazil

RELATO DO ADULTO JOVEM (**18** a **25** anos de idade)

INSTRUÇÕES

A próxima página contém uma lista de coisas com as quais você pode ter dificuldade. Por favor, conte-nos se você **tem tido dificuldade** com alguma dessas coisas durante o **ÚLTIMO MÊS**, fazendo um "X" no número

0 se você **nunca** tem dificuldade com isso
1 se você **quase nunca** tem dificuldade com isso
2 se você **algumas vezes** tem dificuldade com isso
3 se você **muitas vezes** tem dificuldade com isso
4 se você **quase sempre** tem dificuldade com isso

Não existem respostas certas ou erradas.
Caso você não entenda alguma pergunta, por favor, peça ajuda.

PedsQL 4.0 - (18-25) Não pode ser reproduzido sem autorização prévia. Copyright © 1998 JWVarni, Ph.D. Todos os direitos reservados.

PedsQL™ 4.0 Generic Core Scale - Young Adult (18-25) - Brazil/Portuguese - Version of 11 Dec 08 - Mapi Research Institute.
ID4875 / PedsQL-4.0-Core_Yad_AU4.0_por-BR.doc

*Durante o **ÚLTIMO MÊS**, você tem tido **dificuldade** com alguma das coisas abaixo?*

**SOBRE MINHA SAÚDE E MINHAS ATIVIDADES** *(dificuldade para...)*	Nunca	Quase nunca	Algumas vezes	Muitas vezes	Quase sempre
1. Para mim é difícil andar mais de um quarteirão.	0	1	2	3	4
2. Para mim é difícil correr.	0	1	2	3	4
3. Para mim é difícil praticar esportes ou fazer exercícios físicos.	0	1	2	3	4
4. Para mim é difícil levantar coisas pesadas.	0	1	2	3	4
5. Para mim é difícil tomar banho de banheira ou de chuveiro sozinho/a.	0	1	2	3	4
6. Para mim é difícil ajudar nas tarefas domésticas.	0	1	2	3	4
7. Eu sinto dor.	0	1	2	3	4
8. Eu tenho pouca energia ou disposição.	0	1	2	3	4

**SOBRE MEUS SENTIMENTOS** *(dificuldade para...)*	Nunca	Quase nunca	Algumas vezes	Muitas vezes	Quase sempre
1. Eu sinto medo.	0	1	2	3	4
2. Eu me sinto triste.	0	1	2	3	4
3. Eu sinto raiva.	0	1	2	3	4
4. Eu durmo mal.	0	1	2	3	4
5. Eu me preocupo com o que vai acontecer comigo.	0	1	2	3	4

**COMO EU CONVIVO COM OUTRAS PESSOAS** *(dificuldade para...)*	Nunca	Quase nunca	Algumas vezes	Muitas vezes	Quase sempre
1. Eu tenho dificuldade para conviver com outros jovens.	0	1	2	3	4
2. Os outros jovens não querem ser meus amigos.	0	1	2	3	4
3. Os outros jovens implicam comigo.	0	1	2	3	4
4. Eu não consigo fazer coisas que outros jovens da minha idade fazem.	0	1	2	3	4
5. Para mim é difícil acompanhar os jovens da minha idade.	0	1	2	3	4

**SOBRE MEU TRABALHO/MEUS ETUDOS** *(dificuldade para...)*	Nunca	Quase nunca	Algumas vezes	Muitas vezes	Quase sempre
1. É difícil prestar atenção no trabalho ou na aula.	0	1	2	3	4
2. Eu esqueço as coisas.	0	1	2	3	4
3. Eu tenho dificuldade para acompanhar o meu trabalho ou meus estudos.	0	1	2	3	4
4. Eu falto ao trabalho ou à aula por não estar me sentindo bem.	0	1	2	3	4
5. Eu falto ao trabalho ou à aula para ir ao médico ou ao hospital.	0	1	2	3	4

PedsQL 4.0 - (18-25) Não pode ser reproduzido sem autorização prévia. Copyright © 1998 JWVarni, Ph.D. Todos os direitos reservados.

PedsQL™ 4.0 Generic Core Scale - Young Adult (18-25) - Brazil/Portuguese - Version of 11 Dec 08 - Mapi Research Institute.
ID4875 / PedsQL-4.0-Core_Yad_AU4.0_por-BR.doc

N° de identificação: ____________

Data: ______________________

PedsQL™

Questionário pediátrico sobre qualidade de vida

Versão 4.0 – Português (Brasil)

RELATO DO/A ADOLESCENTE (13 a 18 anos)

INSTRUÇÕES

A próxima página contém uma lista de coisas com as quais você pode ter dificuldade.
Por favor, conte-nos se você tem **tido dificuldade** com cada uma dessas coisas durante o **ÚLTIMO MÊS**, fazendo um "X" no número:

0 se você **nunca** tem dificuldade com isso
1 se você **quase nunca** tem dificuldade com isso
2 se você **algumas vezes** tem dificuldade com isso
3 se você **muitas vezes** tem dificuldade com isso
4 se você **quase sempre** tem dificuldade com isso

Não existem respostas certas ou erradas.
Caso você não entenda alguma pergunta, por favor, peça ajuda.

Copyright © 1998 JW Varni, Ph.D. Todos os direitos reservados.
Não pode ser reproduzido sem autorização prévia.

*Durante o **ÚLTIMO MÊS**, você tem tido **dificuldade** com cada uma das coisas abaixo?*

**SOBRE MINHA SAÚDE E MINHAS ATIVIDADES** *(dificuldade para...)*	**Nunca**	**Quase nunca**	**Algumas vezes**	**Muitas vezes**	**Quase sempre**
1. Para mim é difícil andar mais de um quarteirão	0	1	2	3	4
2. Para mim é difícil correr	0	1	2	3	4
3. Para mim é difícil praticar esportes ou fazer exercícios físicos	0	1	2	3	4
4. Para mim é difícil levantar coisas pesadas	0	1	2	3	4
5. Para mim é difícil tomar banho de banheira ou de chuveiro sozinho/a	0	1	2	3	4
6. Para mim é difícil ajudar nas tarefas domésticas	0	1	2	3	4
7. Eu sinto dor	0	1	2	3	4
8. Eu tenho pouca energia ou disposição	0	1	2	3	4

**SOBRE MEUS SENTIMENTOS** *(dificuldade para...)*	**Nunca**	**Quase nunca**	**Algumas vezes**	**Muitas vezes**	**Quase sempre**
1. Eu sinto medo	0	1	2	3	4
2. Eu me sinto triste	0	1	2	3	4
3. Eu sinto raiva	0	1	2	3	4
4. Eu durmo mal	0	1	2	3	4
5. Eu me preocupo com o que vai acontecer comigo	0	1	2	3	4

**COMO EU CONVIVO COM OUTRAS PESSOAS** *(dificuldades para...)*	**Nunca**	**Quase nunca**	**Algumas vezes**	**Muitas vezes**	**Quase sempre**
1. Eu tenho dificuldade para conviver com outros / outras adolescentes	0	1	2	3	4
2. Os outros / as outras adolescentes não querem ser meus amigos / minhas amigas	0	1	2	3	4
3. Os outros / as outras adolescentes implicam comigo	0	1	2	3	4
4. Eu não consigo fazer coisas que outros / outras adolescentes da minha idade fazem	0	1	2	3	4
5. Para mim é difícil acompanhar os / as adolescentes da minha idade	0	1	2	3	4

**SOBRE A ESCOLA** *(dificuldades para...)*	**Nunca**	**Quase nunca**	**Algumas vezes**	**Muitas vezes**	**Quase sempre**
1. É difícil prestar atenção na aula	0	1	2	3	4
2. Eu esqueço as coisas	0	1	2	3	4
3. Eu tenho dificuldade para acompanhar a minha turma nas tarefas escolares	0	1	2	3	4
4. Eu falto à aula por não estar me sentindo bem	0	1	2	3	4
5. Eu falto à aula para ir ao médico ou ao hospital	0	1	2	3	4

Copyright © 1998 JW Varni, Ph.D. Todos os direitos reservados.
Não pode ser reproduzido sem autorização prévia.

N° de identificação: ___________

Data: ___________________

PedsQL™

Questionário pediátrico sobre qualidade de vida

Versão 4.0 – Português (Brasil)

RELATO DA CRIANÇA (8 a 12 anos)

INSTRUÇÕES

A próxima página contém uma lista de coisas com as quais você pode ter dificuldade.
Por favor, conte-nos se você **tem tido dificuldade** com cada uma dessas coisas durante o **ÚLTIMO MÊS**, fazendo um "X" no número:

0 se você **nunca** tem dificuldade com isso
1 se você **quase nunca** tem dificuldade com isso
2 se você **algumas vezes** tem dificuldade com isso
3 se você **muitas vezes** tem dificuldade com isso
4 se você **quase sempre** tem dificuldade com isso

Não existem respostas certas ou erradas.
Caso você não entenda alguma pergunta, por favor, peça ajuda.

Copyright © 1998 JW Varni, Ph.D. Todos os direitos reservados.
Não pode ser reproduzido sem autorização prévia.

Durante o ÚLTIMO MÊS, você tem tido dificuldade com cada uma das coisas abaixo?

**SOBRE MINHA SAÚDE E MINHAS ATIVIDADES** *(dificuldade para...)*	**Nunca**	**Quase nunca**	**Algumas vezes**	**Muitas vezes**	**Quase sempre**
1. Para mim é difícil andar mais de um quarteirão	0	1	2	3	4
2. Para mim é difícil correr	0	1	2	3	4
3. Para mim é difícil praticar esportes ou fazer exercícios físicos	0	1	2	3	4
4. Para mim é difícil levantar coisas pesadas	0	1	2	3	4
5. Para mim é difícil tomar banho de banheira ou de chuveiro sozinho/a	0	1	2	3	4
6. Para mim é difícil ajudar nas tarefas domésticas	0	1	2	3	4
7. Eu sinto dor	0	1	2	3	4
8. Eu me sinto cansado/a	0	1	2	3	4

**SOBRE MEUS SENTIMENTOS** *(dificuldade para...)*	**Nunca**	**Quase nunca**	**Algumas vezes**	**Muitas vezes**	**Quase sempre**
1. Eu sinto medo	0	1	2	3	4
2. Eu me sinto triste	0	1	2	3	4
3. Eu sinto raiva	0	1	2	3	4
4. Eu durmo mal	0	1	2	3	4
5. Eu me preocupo com o que vai acontecer comigo	0	1	2	3	4

**COMO EU CONVIVO COM OUTRAS PESSOAS** *(dificuldades para...)*	**Nunca**	**Quase nunca**	**Algumas vezes**	**Muitas vezes**	**Quase sempre**
1. Eu tenho dificuldade para conviver com outras crianças	0	1	2	3	4
2. As outras crianças não querem ser minhas amigas	0	1	2	3	4
3. As outras crianças implicam comigo	0	1	2	3	4
4. Eu não consigo fazer coisas que outras crianças da minha idade fazem	0	1	2	3	4
5. Para mim é difícil acompanhar a brincadeira com outras crianças	0	1	2	3	4

**SOBRE A ESCOLA** *(dificuldades para...)*	**Nunca**	**Quase nunca**	**Algumas vezes**	**Muitas vezes**	**Quase sempre**
1. É difícil prestar atenção na aula	0	1	2	3	4
2. Eu esqueço as coisas	0	1	2	3	4
3. Eu tenho dificuldade para acompanhar a minha turma nas tarefas escolares	0	1	2	3	4
4. Eu falto à aula por não estar me sentindo bem	0	1	2	3	4
5. Eu falto à aula para ir ao médico ou ao hospital	0	1	2	3	4

Copyright © 1998 JW Varni, Ph.D. Todos os direitos reservados.
Não pode ser reproduzido sem autorização prévia.

Nº de identificação: ____________
Data: ____________

PedsQL™

Questionário pediátrico sobre qualidade de vida

Versão 4.0 – Portuguese (Brazil)

RELATO DA CRIANÇA (5 a 7 anos)

Instruções para o entrevistador:

Eu vou te fazer algumas perguntas sobre coisas que podem ser difíceis para algumas crianças. Eu quero saber se cada uma dessas coisas pode ser difícil para você.

Mostre à criança a página com as carinhas e conforme você for lendo as frases abaixo aponte a resposta correspondente.

Se isso nunca é difícil, aponte a carinha sorridente.

Se isso algumas vezes é difícil, aponte a carinha do meio.

Se isso quase sempre é difícil, aponte a carinha zangada.

Eu vou ler as perguntas uma por uma. Quando eu acabar de ler uma pergunta, você vai apontar a resposta para me dizer se isso é difícil para você. Vamos treinar primeiro.

	Nunca	Algumas vezes	Quase sempre
**Para você é difícil estalar os dedos?**	☺	😐	☹

Para determinar se a criança respondeu corretamente à pergunta ou não, peça-lhe que mostre como estala os dedos. Repita a pergunta se a criança mostrou uma resposta diferente da ação.

Copyright © 1998 JW Varni, Ph.D. Todos os direitos reservados.
Não pode ser reproduzido sem autorização prévia.

Pense em como você tem se sentido durante as últimas semanas. Por favor, escute cada uma das frases com bastante atenção e me conte se cada uma destas coisas é difícil para você.

Depois de ler o item mostre à criança a página com as carinhas. Se ela hesitar ou parecer não saber como responder, leia as opções de resposta enquanto aponta as carinhas.

**CAPACIDADE FÍSICA** *(é difícil...)*	**Nunca**	**Algumas vezes**	**Quase sempre**
1. Você acha difícil andar?	0	2	4
2. Você acha difícil correr?	0	2	4
3. Você acha difícil fazer exercícios físicos ou esportes?	0	2	4
4. Você acha difícil levantar coisas pesadas?	0	2	4
5. Você acha difícil tomar banho de banheira ou de chuveiro?	0	2	4
6. Você acha difícil ajudar nas tarefas domésticas (como apanhar os seus brinquedos)?	0	2	4
7. Você sente dor? (*Onde?*______________)	0	2	4
8. Você se sente cansado/a demais para brincar?	0	2	4

Lembre-se, você vai me contar se isto tem sido difícil para você durante as últimas semanas.

**ASPECTO EMOCIONAL** *(é difícil...)*	**Nunca**	**Algumas vezes**	**Quase sempre**
1. Você sente medo?	0	2	4
2. Você se sente triste?	0	2	4
3. Você sente raiva?	0	2	4
4. Você dorme mal?	0	2	4
5. Você se preocupa com que vai acontecer com você?	0	2	4

**ASPECTO SOCIAL** *(é difícil...)*	**Nunca**	**Algumas vezes**	**Quase sempre**
1. Você acha difícil conviver com outras crianças?	0	2	4
2. As outras crianças dizem que não querem brincar com você?	0	2	4
3. As outras crianças implicam com você?	0	2	4
4. As outras crianças fazem coisas que você não consegue fazer?	0	2	4
5. Você acha difícil acompanhar as brincadeiras com outras crianças?	0	2	4

Copyright © 1998 JW Varni, Ph.D. Todos os direitos reservados.
Não pode ser reproduzido sem autorização prévia.

ATIVIDADE ESCOLAR *(é difícil...)*	Nunca	Algumas vezes	Quase sempre
1. Você acha difícil prestar atenção na aula?	0	2	4
2. Você esquece as coisas?	0	2	4
3. Você acha difícil acompanhar a sua turma nas tarefas escolares?	0	2	4
4. Você falta à aula porque você não se sente bem?	0	2	4
5. Você falta à aula porque você tem que ir ao médico ou ao hospital?	0	2	4

Copyright © 1998 JW Varni, Ph.D. Todos os direitos reservados.
Não pode ser reproduzido sem autorização prévia.

Bowel Function Score

Factor	Score Given
Ability to hold back defecation	
Always	3
Problems <1/week	2
Weekly problems	1
No voluntary control	0
Feels the urge to defecate	
Always	3
Most of the time	2
Uncertain	1
Absent	0
Frequency of defecation	
Every other day—twice a day	2
More often	1
Less often	1
Soiling	
Never	3
Staining <1/week, no change of underwear required	2
Frequent staining/soiling, change of underwear required	1
Daily soiling, requires protective aids	0
Accidents	
Never	3
Less than 1/week	2
Weekly accidents, often requires protective aids	1
Daily, protective aids required day and night	0
Constipation	
No constipation	3
Manageable with diet	2
Manageable with laxatives	1
Manageable with enemas	0
Social problems	
No social problems	3
Sometimes (foul odors)	2
Problems causing restrictions of social life	1
Major social/psychological problems	0

Adapted from
Jarvi K, Laitakari EM, Koivusalo A, Rintala RJ, Pakarinen MP. Bowel function and gastrointestinal quality of life among adults operated for Hirschsprung disease during childhood: a population-based study. Ann Surg. 2010; 252(6):977-981.

SPIRIT
STANDARD PROTOCOL ITEMS: RECOMMENDATIONS FOR INTERVENTIONAL TRIALS

SPIRIT 2013 Checklist: Recommended items to address in a clinical trial protocol and related documents*

Section/item	Item No	Description	Addressed on page number
**Administrative information**			
Title	1	Descriptive title identifying the study design, population, interventions, and, if applicable, trial acronym	______1______
Trial registration	2a	Trial identifier and registry name. If not yet registered, name of intended registry	______1______
	2b	All items from the World Health Organization Trial Registration Data Set	______4______
Protocol version	3	Date and version identifier	______1______
Funding	4	Sources and types of financial, material, and other support	______1______
Roles and responsibilities	5a	Names, affiliations, and roles of protocol contributors	______1______
	5b	Name and contact information for the trial sponsor	_____NA______
	5c	Role of study sponsor and funders, if any, in study design; collection, management, analysis, and interpretation of data; writing of the report; and the decision to submit the report for publication, including whether they will have ultimate authority over any of these activities	_____NA______
	5d	Composition, roles, and responsibilities of the coordinating centre, steering committee, endpoint adjudication committee, data management team, and other individuals or groups overseeing the trial, if applicable (see Item 21a for data monitoring committee)	_____NA______

Introduction

Background and rationale	6a	Description of research question and justification for undertaking the trial, including summary of relevant studies (published and unpublished) examining benefits and harms for each intervention	_____3,4_____
	6b	Explanation for choice of comparators	______3,4____
Objectives	7	Specific objectives or hypotheses	______4_____
Trial design	8	Description of trial design including type of trial (eg, parallel group, crossover, factorial, single group), allocation ratio, and framework (eg, superiority, equivalence, noninferiority, exploratory)	______4_____

Methods: Participants, interventions, and outcomes

Study setting	9	Description of study settings (eg, community clinic, academic hospital) and list of countries where data will be collected. Reference to where list of study sites can be obtained	_______4____
Eligibility criteria	10	Inclusion and exclusion criteria for participants. If applicable, eligibility criteria for study centres and individuals who will perform the interventions (eg, surgeons, psychotherapists)	__5, table 1_____
Interventions	11a	Interventions for each group with sufficient detail to allow replication, including how and when they will be administered	_______5,6____
	11b	Criteria for discontinuing or modifying allocated interventions for a given trial participant (eg, drug dose change in response to harms, participant request, or improving/worsening disease)	______N/A____
	11c	Strategies to improve adherence to intervention protocols, and any procedures for monitoring adherence (eg, drug tablet return, laboratory tests)	______N/A_____
	11d	Relevant concomitant care and interventions that are permitted or prohibited during the trial	______N/A____
Outcomes	12	Primary, secondary, and other outcomes, including the specific measurement variable (eg, systolic blood pressure), analysis metric (eg, change from baseline, final value, time to event), method of aggregation (eg, median, proportion), and time point for each outcome. Explanation of the clinical relevance of chosen efficacy and harm outcomes is strongly recommended	______6______
Participant timeline	13	Time schedule of enrolment, interventions (including any run-ins and washouts), assessments, and visits for participants. A schematic diagram is highly recommended (see Figure)	__5,6,Figure 4___

Sample size	14	Estimated number of participants needed to achieve study objectives and how it was determined, including clinical and statistical assumptions supporting any sample size calculations	_______5____
Recruitment	15	Strategies for achieving adequate participant enrolment to reach target sample size	_______5_____

Methods: Assignment of interventions (for controlled trials)

Allocation:			
Sequence generation	16a	Method of generating the allocation sequence (eg, computer-generated random numbers), and list of any factors for stratification. To reduce predictability of a random sequence, details of any planned restriction (eg, blocking) should be provided in a separate document that is unavailable to those who enrol participants or assign interventions	____N/A______
Allocation concealment mechanism	16b	Mechanism of implementing the allocation sequence (eg, central telephone; sequentially numbered, opaque, sealed envelopes), describing any steps to conceal the sequence until interventions are assigned	____N/A______
Implementation	16c	Who will generate the allocation sequence, who will enrol participants, and who will assign participants to interventions	____N/A______
Blinding (masking)	17a	Who will be blinded after assignment to interventions (eg, trial participants, care providers, outcome assessors, data analysts), and how	____N/A______
	17b	If blinded, circumstances under which unblinding is permissible, and procedure for revealing a participant's allocated intervention during the trial	____N/A_____

Methods: Data collection, management, and analysis

Data collection methods	18a	Plans for assessment and collection of outcome, baseline, and other trial data, including any related processes to promote data quality (eg, duplicate measurements, training of assessors) and a description of study instruments (eg, questionnaires, laboratory tests) along with their reliability and validity, if known. Reference to where data collection forms can be found, if not in the protocol	_____5,6,7_____
	18b	Plans to promote participant retention and complete follow-up, including list of any outcome data to be collected for participants who discontinue or deviate from intervention protocols	_____5,6_____

Data management	19	Plans for data entry, coding, security, and storage, including any related processes to promote data quality (eg, double data entry; range checks for data values). Reference to where details of data management procedures can be found, if not in the protocol	_____5,6_____
Statistical methods	20a	Statistical methods for analysing primary and secondary outcomes. Reference to where other details of the statistical analysis plan can be found, if not in the protocol	______7______
	20b	Methods for any additional analyses (eg, subgroup and adjusted analyses)	______7_______
	20c	Definition of analysis population relating to protocol non-adherence (eg, as randomised analysis), and any statistical methods to handle missing data (eg, multiple imputation)	______N/A______
**Methods: Monitoring**			
Data monitoring	21a	Composition of data monitoring committee (DMC); summary of its role and reporting structure; statement of whether it is independent from the sponsor and competing interests; and reference to where further details about its charter can be found, if not in the protocol. Alternatively, an explanation of why a DMC is not needed	______N/A______
	21b	Description of any interim analyses and stopping guidelines, including who will have access to these interim results and make the final decision to terminate the trial	_______N/A_____
Harms	22	Plans for collecting, assessing, reporting, and managing solicited and spontaneously reported adverse events and other unintended effects of trial interventions or trial conduct	_______N/A_____
Auditing	23	Frequency and procedures for auditing trial conduct, if any, and whether the process will be independent from investigators and the sponsor	_______N/A_____
**Ethics and dissemination**			
Research ethics approval	24	Plans for seeking research ethics committee/institutional review board (REC/IRB) approval	_______4______
Protocol amendments	25	Plans for communicating important protocol modifications (eg, changes to eligibility criteria, outcomes, analyses) to relevant parties (eg, investigators, REC/IRBs, trial participants, trial registries, journals, regulators)	______7_______

Consent or assent	26a	Who will obtain informed consent or assent from potential trial participants or authorised surrogates, and how (see Item 32)	________4______
	26b	Additional consent provisions for collection and use of participant data and biological specimens in ancillary studies, if applicable	________4_____
Confidentiality	27	How personal information about potential and enrolled participants will be collected, shared, and maintained in order to protect confidentiality before, during, and after the trial	________4______
Declaration of interests	28	Financial and other competing interests for principal investigators for the overall trial and each study site	________1______
Access to data	29	Statement of who will have access to the final trial dataset, and disclosure of contractual agreements that limit such access for investigators	________7_____
Ancillary and post-trial care	30	Provisions, if any, for ancillary and post-trial care, and for compensation to those who suffer harm from trial participation	_______N/A______
Dissemination policy	31a	Plans for investigators and sponsor to communicate trial results to participants, healthcare professionals, the public, and other relevant groups (eg, via publication, reporting in results databases, or other data sharing arrangements), including any publication restrictions	_______7_______
	31b	Authorship eligibility guidelines and any intended use of professional writers	_______1,7______
	31c	Plans, if any, for granting public access to the full protocol, participant-level dataset, and statistical code	_______7_______
**Appendices**			
Informed consent materials	32	Model consent form and other related documentation given to participants and authorised surrogates	__supplemental digital content 2 and 3______
Biological specimens	33	Plans for collection, laboratory evaluation, and storage of biological specimens for genetic or molecular analysis in the current trial and for future use in ancillary studies, if applicable	___N/A_______

*It is strongly recommended that this checklist be read in conjunction with the SPIRIT 2013 Explanation & Elaboration for important clarification on the items. Amendments to the protocol should be tracked and dated. The SPIRIT checklist is copyrighted by the SPIRIT Group under the Creative Commons "Attribution-NonCommercial-NoDerivs 3.0 Unported" license.
